# Mitochondrial characteristics of echinocandin tolerance and resistance in *Candida glabrata* and *Candida krusei*

**DOI:** 10.3389/fmicb.2026.1906168

**Published:** 2026-07-27

**Authors:** Peng Xue, Li Zhang, Mingwei Du, Quan Yuan, Haiyang He, Xiaochun Xue, Wanqing Liao, Yuanyuan Ma, Weihua Pan, Liuyang Cai

**Affiliations:** 1 School of Public Health, Nantong University, Nantong, China; 2 Department of Dermatology, Shanghai Key Laboratory of Molecular Medical Mycology, Shanghai Changzheng Hospital, Naval Medical University, Shanghai, China; 3 The Center for Fungal Infectious Diseases Basic Research and Innovation of Medicine and Pharmacy, Ministry of Education, Shanghai, China; 4 Oriental Institute of Infection and Inflammation, Guangzhou, China; 5 Department of Pharmacy, 905th Hospital of People’s Liberation Army Navy, Shanghai, China

**Keywords:** *Candida glabrata*, *Candida krusei*, echinocandin resistance, echinocandin tolerance, mitochondrial characteristics

## Abstract

**Background:**

Candidiasis, especially candidemia, is one of the most common invasive fungal infections. With the increase of risk factors such as tumors, its high incidence and high mortality rate pose a significant challenge to public health. Echinocandins, as first-line drugs for candidemia, have their clinical efficacy severely weakened by the emergence of tolerant or resistant strains such as *Candida glabrata* and *Candida krusei*.

**Methods:**

The study evaluated the changes in mitochondrial phenotype (intracellular ATP levels, mitochondrial membrane potential, mitochondrial superoxide levels, and intracellular ROS levels), proteomic, metabolomic, and genomic in *C. glabrata* and *C. krusei*.

**Results:**

By analyzing strains with different susceptibility profiles, the study found that echinocandin tolerant or resistant strains displayed significant upregulation of ATP levels and unique metabolic adaptations. Quantitative proteomics identified differentially expressed proteins associated with mitochondrial function and energy metabolism. Metabolomic analysis further revealed distinct profiles linking resistance to alterations in purine metabolism and oxidative phosphorylation. Additionally, genomic sequencing of the resistant strains highlighted mutations in key genes involved in ATP binding.

**Conclusion:**

These findings highlight the mitochondrial characteristics in echinocandin tolerance and resistance in *C. glabrata* and *C. krusei*, providing valuable insights into potential pathways for future therapeutic interventions targeting resistant *Candida* species.

## 1 Introduction

Candidemia is an invasive candidiasis caused by the entry of *Candida* into the bloodstream and is often associated with a poor prognosis (Lass-Flörl et al., 2024). The increase of high-risk patients, the suitable environment for fungal growth created by global warming, and the frequent communication and interaction of human beings have jointly led to the rise in the incidence of candidemia and the expansion of the geographical scope of the impact (Ricotta et al., 2021). The pathogen spectrum has also changed significantly, and the proportion of non-*Candida albicans*, especially *Candida glabrata* and *Candida krusei* has increased (Xiao et al., 2020; Bilal et al., 2025).

Currently, there are only four categories of systemic antifungal drugs available. However, the widespread application of pesticides in the environment and antifungal drugs in clinics has aggravated the adaptive evolution of *Candida* tolerance or resistance, further increasing the medical burden and mortality risk (Fisher et al., 2022). As widely used antifungal drugs, azoles and flucytosine face troubles in clinical application due to the growing prevalence of cross-resistance (Arastehfar et al., 2020; Bilal et al., 2022). Although polyene antifungals, such as amphotericin B have a broad spectrum of activity and low resistant rates, their clinical use is limited by severe adverse reactions such as nephrotoxicity and hepatotoxicity (Pappas et al., 2016). Echinocandins have become the first-choice antifungal drugs for the treatment of candidemia due to their high efficiency and low toxicity (Thompson et al., 2024), but the emergence of echinocandin tolerant or resistant strains such as *C. glabrata* and *C. krusei* has seriously weakened its clinical efficacy (Paiva and Pereira, 2023). In conclusion, the fungal infection represented by candidemia poses a serious threat to public health, and the drug tolerance or resistance of *Candida* has become a new and major crisis for the global antifungal treatment. It is urgent to study the tolerant or resistant mechanism that affects the sensitivity change of *Candida* to antifungal drugs, especially echinocandins, in order to prevent and control the evolution of drug resistance and improve the clinical prognosis.

Previously, it was believed that *Candida* resistant mechanisms were primarily driven by genetic mutations, such as point mutations in target genes, gene amplification, or upregulation of efflux pumps (Fisher et al., 2022). Echinocandin resistance in *Candida* is generally associated with the gene mutations in specific hotspot (HS) regions of *FKS1* and *FKS2*, which encode echinocandin target *β*-1, 3-D-glucan synthase (GS) (Perlin, 2015). However, in fact, after the failure of echinocandin treatment in some clinical cases of candidemia, the isolates did not detect mutations in known classical resistant genes such as *FKS1* and *FKS2*, suggesting that there may be non-gene driven resistant mechanisms (Daneshnia et al., 2026).

Mitochondria play a critical role in energy metabolism, cellular stress responses, and programmed cell death, and emerging evidence suggests their involvement in antifungal resistant mechanisms (Zhou et al., 2024; Ma et al., 2025; Zhang et al., 2025). The relationship between mitochondrial function and drug resistance in fungi has become more apparent, as mitochondria regulate various metabolic pathways that enhance a cell’s resilience to antifungal agents (Shingu-Vazquez and Traven, 2011; Ma et al., 2025). Changes in mitochondrial activity can result in alterations in ATP production, reactive oxygen species (ROS) generation, and overall energy balance, potentially conferring a selective advantage under antifungal exposure (Zhou et al., 2024). Research has indicated that drug resistance in *C. glabrata* arises from both genetic mutations and metabolic adaptations, particularly those linked to mitochondrial function (Garcia-Rubio et al., 2021). For instance, azole resistance in some *Candida* species, including *C. albicans*, *C. glabrata*, and *Candida auris*, has been associated with specific mitochondrial activities and distinct metabolic pathways, with azole-resistant strains exhibiting elevated ATP levels. Furthermore, proteomic analyses of mitochondria across these *Candida* species have revealed unique protein profiles that differentiate between azole-sensitive and resistant strains (Zhou et al., 2024).

Therefore, the study aims to provide a comprehensive description of the mitochondrial characteristics of echinocandin tolerant and resistant *C. glabrata* and *C. krusei* through the changes of mitochondrial phenotype, proteomic, metabolomic, and genomic levels.

## 2 Materials and methods

### 2.1 Strain and media

This study utilized different characteristics of *C. glabrata*, including a standard strain CBS138 with echinocandin susceptibility, a clinical isolate #21 with echinocandin tolerant, and two experimental isolates with echinocandin resistant ECR60 and ECR157. CBS138, #21, and ECR60 all without *FKS* mutation, while ECR157 has a classic *FKS* mutation F659del (*FKS* sequence of four *C. glabrata*, please see Supplementary Data 1). All strains have been confirmed to be correct through Sanger sequencing to identify ITS and Blast (Supplementary Data 2). Specifically, ECR60 shows caspofungin reduced susceptibility but micafungin increased susceptibility (CRS-MIS) phenotype. However, the MIC of ECR60 to caspofungin is 1.00 μg/mL, indicating resistance to caspofungin. ECR60 still belongs to an echinocandin resistant *C. glabrata*. In contrast, the MIC of ECR157 for caspofungin and micafungin are 8.00 and 1.00 μg/mL, respectively, indicating resistance to both caspofungin and micafungin (Supplementary Data 3). CBS138 and #21 are both sensitive to echinocandin and have similar minimum inhibitory concentrations, but experiments have shown that CBS138 shows low echinocandin tolerant and does not induce echinocandin resistant progenies, while #21 shows high echinocandin tolerant (can grow at concentrations of echinocandin higher than the minimum inhibitory concentration) and can induce echinocandin resistant progenies (Arastehfar et al., 2021; Arastehfar et al., 2024). ECR60 and ECR157 are both induced and isolated by continuous stimulation of echinocandin in animal infected with #21 (Cai et al., 2026). Specifically, six-week-old female C57BL/6 mice were intravenously infected with 100 μL of a suspension containing 2 × 10^7^ CFU/mL of #21. After infection, the mice received intraperitoneal injections of caspofungin at a dosage of 5 mg/kg every two days. Tissue samples from the liver, spleen, and kidneys were collected at designated time points (Days 6, 8, 10, 12, 14, 16, 18, and 20) for further analysis. The ECR157 was isolated from spleen tissue on Day 12, while the ECR60 was obtained from spleen tissue on Day 20. To isolate drug-resistant strains, the harvested tissues were homogenized and inoculated onto YPD agar plates (2% dextrose, 2% peptone, 1% yeast extract, and 2% agar), both with and without 0.25 μg/mL of caspofungin. A 10-pass subculture in drug-free media was subsequently performed to ensure stable resistance. In addition to the aforementioned *C. glabrata* strains, we included the standard strain *C. krusei* (ATCC6258) along with clinical isolate CK225 to evaluate the universality of mitochondrial changes across different *Candida* species. Antifungal susceptibility testing of all strains was performed according to the Clinical and Laboratory Standards Institute and the European Committee on Antimicrobial Susceptibility Testing (Supplementary Data 3) (CLSI, 2022; EUCAST, 2024).

*C. glabrata* (recently reclassified as *Nakaseomyces glabrata*) and *C. krusei* (recently reclassified as *Pichia kudriavzevii*) were used in this study, with the former names retained for clinical clarity and consistency with previous literature. The strains isolated from previous animal experiments used in this study were obtained under an original animal experimental protocol by the Naval Medical University (Ethics Committee approval no. 82204470). All experimental procedures were strictly performed in accordance with the guidelines for the care and use of laboratory animals. The clinical isolates used in this study were obtained from Jiangxi Cancer Hospital (Ethics Committee approval no.: MR-36-24-034076,). Since no identifiable patient information was involved, the committee waived the requirement for informed consent.

### 2.2 Measurement of intracellular ATP levels, mitochondrial membrane potential, mitochondrial superoxide, and intracellular ROS

Fungal cells were cultured overnight at 130 rpm and 30 °C in YPD medium, followed by two washes with PBS. After treatment, we quantified intracellular ATP levels using the BacTiter-Glo Microbial Cell Viability Assay kit (Promega, United States), with signal detection performed on a microplate reader (TECAN Infinite E Plex). For mitochondrial membrane potential assessment, cells were stained with 50 nM Mito-Tracker Red CMXRos. Mito-SOX Red (50 μM) was used to detect mitochondrial superoxide, while 2′,7′-Dichlorofluorescein Diacetate (DCFH-DA, 16 μM) measured intracellular ROS levels. All staining procedures were conducted at 30 °C for 1 h. The fluorescence intensity of the stained cells was measured using the multimode microplate reader (TECAN Infinite E Plex). The experiment was replicated three times.

### 2.3 Proteomic analysis

Frozen cells were ground in liquid nitrogen and lysed in a buffer containing protease and phosphatase inhibitors. Proteins were extracted via phenol phase separation, precipitated with methanol, and quantified using a BCA assay. After reduction, alkylation, and tryptic digestion, the resulting peptides were desalted and analyzed by LC–MS/MS on a timsTOF Pro mass spectrometer coupled to an Easy-nLC1000 system. Raw data were processed using DIA-NN against organism-specific databases. Functional annotations (Gene Ontology (GO), KEGG pathways, and subcellular localization) were assigned, and differentially expressed proteins were identified based on statistical enrichment analysis. Detailed technical parameters, including buffer compositions, gradient settings, database search criteria, and enrichment thresholds, are provided in the Supplementary Material.

### 2.4 Metabolomic profiling

For metabolomic profiling, cell samples that had been frozen at −80 °C were initially thawed on ice. They were then homogenized for 20 s using a grinder. The resultant homogenates were combined with 400 μL of a 4:1 (v/v) methanol–water solution containing an internal standard and vortexed for three minutes. To enhance metabolite extraction, the mixture was subjected to three freeze–thaw cycles using liquid nitrogen and dry ice, with vortexing following each cycle. Cellular debris was removed via centrifugation at 12,000 rpm for 10 min at 4 °C, and 300 μL of the supernatant was collected and stored at −20 °C for 30 min prior to a secondary centrifugation. For LC–MS analysis, a 200 μL aliquot from the supernatant was extracted. Each sample underwent analysis using two complementary LC–MS methodologies.

The first method employed a positive ionization mode utilizing a T3 column (Waters ACQUITY Premier HSS T3, 2.1 mm × 100 mm, 1.8 μm). Mobile phase A consisted of 0.1% formic acid in water, while phase B was 0.1% formic acid in acetonitrile. The elution gradient initiated at 5% B, ramping to 20% B over 2 min, followed by an increase to 60% B over the subsequent 3 min, a rapid ascent to 99% B within 1 min, maintained for an additional 1.5 min before reverting to 5% B in 0.1 min, with re-equilibration for 2.4 min. Column temperature was sustained at 40 °C, with a flow rate of 0.4 mL/min and an injection volume of 4 μL.

The second method utilized negative ionization with an identical elution gradient. Data acquisition was carried out in information-dependent acquisition (IDA) mode and processed using Analyst TF 1.7.1 software (Sciex). Key ion source parameters were set as follows: Ion source gas 1 and 2 at 50 psi, curtain gas at 25 psi, and a source temperature of 550 °C. Declustering potentials were adjusted to 60 V for positive and −60 V for negative modes, with ion spray voltages at floating values of 5,000 V and −4,000 V, respectively. TOF MS scans were executed over a mass range of 50–1,000 Da, with a 200 ms accumulation time. Product ion scans spanned 25–1,000 Da with a 40 ms accumulation time, 30 V (positive) and −30 V (negative) collision energies, and a collision energy spread of 15, all at UNIT resolution. The instrument monitored singly charged ions with an intensity threshold of 100 cps, excluding isotopes within 4 Da, implementing a mass tolerance of 50 ppm, and targeting a maximum of 18 candidates per cycle. The raw LC–MS data were converted to mzXML format using ProteoWizard. Procedures for peak extraction, alignment, and retention time correction were performed using the XCMS program, with peaks detected in less than 50% of samples discarded.

Metabolite identification was facilitated through referencing the laboratory’s custom and public resources, such as HMDB, Metlin, MassBank, Mona, KEGG, as well as AI databases and metDNA. Metabolites exhibiting significant differences were identified through a combination of Variable Importance in Projection (VIP) scores exceeding 1 from Orthogonal Partial Least Squares Discriminant Analysis (OPLS-DA) and *p*-values below 0.05 from Student’s t-test. Finally, KEGG pathway enrichment analyses were conducted for differentially expressed proteins and metabolites.

### 2.5 Genomic analysis

Genomic DNA was extracted from fungal cell samples using the NucleoBond^®^ HMW DNA extraction kit (MN NucleoBond, Germany, 740160.20). DNA concentration and purity were evaluated using a Qubit 4.0 fluorometer (Thermo, Q33226), and integrity was confirmed by 0.75% agarose gel electrophoresis. For library preparation, genomic DNA was randomly fragmented to an average size of 200–400 bp, followed by end-repair, 3′ adenylation, adapter ligation, and PCR amplification. Libraries were purified with magnetic beads, quantified with Qubit 4.0, and size-checked on 2% agarose gels. Qualified libraries were sequenced on the MGI DNBSEQ-T7 platform at Sangon Biotech (Shanghai, China), generating 300 bp paired-end reads.

Raw reads were quality-controlled using Trimmomatic (v0.39) (Bolger et al., 2014) to remove adapters, low-quality bases (Q < 20), and reads shorter than 35 bp. The cleaned reads (clean data) were then aligned to the reference genome using BWA-MEM (v0.7.17). SAMtools (v1.9) (Li et al., 2009) was used to sort and convert alignment files, and duplicate reads were marked with GATK’s MarkDuplicates (v4.1.1.0) (McKenna et al., 2010). Single nucleotide polymorphisms (SNPs) and small insertions/deletions (InDels) were detected using GATK HaplotypeCaller, following the best practice workflow. Variant calls were filtered to retain those with read depth ≥ 4 and mapping quality ≥ 30. The effects of variants were annotated with SnpEff (v4.3) (Cingolani et al., 2012) using the reference gene annotation. Only genes affected by non-synonymous (missense) variants, frameshift variants, stop-gain/loss, and splice-region variants were retained as “variant-affected genes” for downstream functional enrichment.

GO enrichment analysis was performed using the topGO R package (v2.36.0) with the “weight01” algorithm and a Fisher’s exact test. The background gene set comprised all protein-coding genes in the reference genome. Multiple testing was corrected by the Benjamini-Hochberg false discovery rate (FDR), and GO terms with an adjusted *p*-value (q-value) < 0.05 were considered significantly enriched. Enriched Molecular Function (MF) terms were visualized as a bubble plot, where the rich factor, q-value, and the number of associated genes (Signi Num) are indicated for each term. The significantly enriched GO terms from all test samples were intersected to identify commonly enriched functional categories, and the MF-term bubble plot was generated based on the union of enriched terms.

## 3 Results

### 3.1 Comparative proteomics and metabolomics of echinocandin susceptible *Candida glabrata* vs. echinocandin tolerant *Candida glabrata* (CBS138 vs. #21)

**Figure 1:**
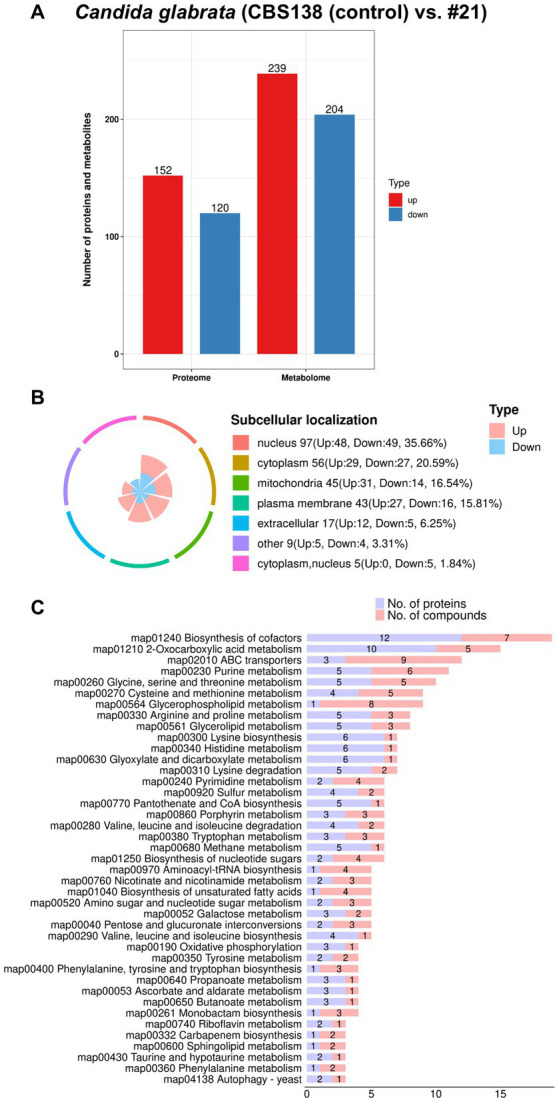
Comparison of protein and metabolite profiles between the echinocandin susceptible standard strain CBS138 and the echinocandin tolerant clinical isolate #21 of *Candida glabrata*. **(A)** Bar charts depict differentially expressed proteins and metabolites: up-regulated proteins are shown in red with a ratio > 1.5 and *p* < 0.05, down-regulated proteins in blue with a ratio < 0.67 and *p* < 0.05, and metabolites with VIP > 1 and *p* < 0.05. **(B)** Subcellular localization of the identified differentially expressed proteins is included. **(C)** Pathway-based classification and correlation analyses of the differentially expressed metabolites and proteins.

**Figure 2:**
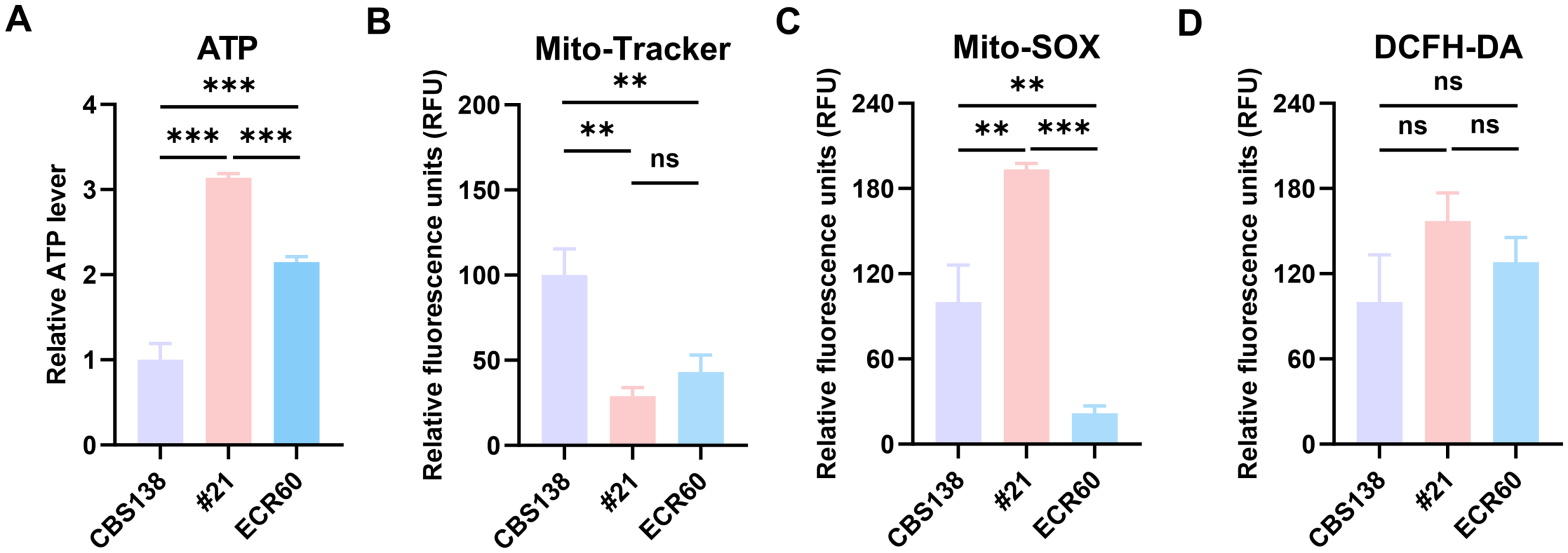
Assessment of intracellular parameters among three *Candida glabrata* strains: the echinocandin susceptible strain CBS138, the echinocandin tolerant isolate #21, and the echinocandin resistant isolate ECR60. **(A)** Comparison of intracellular ATP levels. **(B)** Evaluation of mitochondrial membrane potential. **(C)** Measurement of mitochondrial superoxide levels. **(D)** Analysis of intracellular ROS levels. Statistical significance was determined using a t-test, with results indicated as ** (*p* < 0.01), *** (*p* < 0.001), and ns for not significant.

We compared the proteomic and metabolomic characterization of the echinocandin susceptible standard strain CBS138 with the echinocandin tolerant clinical isolate #21 (Supplementary Data 3) (Arastehfar et al., 2021; Arastehfar et al., 2024). Quantitative proteomic analysis disclosed that 152 proteins were upregulated while 120 were downregulated in the #21 (Figure 1A). Untargeted metabolomics similarly identified 239 upregulated metabolites and 204 downregulated metabolites (Figure 1B; Supplementary Data 4). Mitochondrial protein analysis revealed notable changes, identifying 45 proteins with alterations, including 31 upregulated and 14 downregulated (Supplementary Table S1). Correlation analysis between differentially expressed proteins and metabolites established connections to oxidative phosphorylation and purine metabolism (Figure 1C), demonstrating a strong relationship to ATP generation. Significant differences in ATP and mitochondrial ROS levels were observed between echinocandin susceptible and echinocandin tolerant strains (CBS138 vs. #21). While the membrane potential was lower in the tolerant strain, intracellular ROS levels did not differ significantly (Figure 2). Overall, these results reveal the mitochondrial characteristics of echinocandin tolerant in *C. glabrata*.

### 3.2 Comparative proteomics and metabolomics of echinocandin tolerant *Candida glabrata* vs. echinocandin resistant *Candida glabrata* with CRS-MIS phenotype and no *FKS* mutation (#21 vs. ECR60)

**Figure 3:**
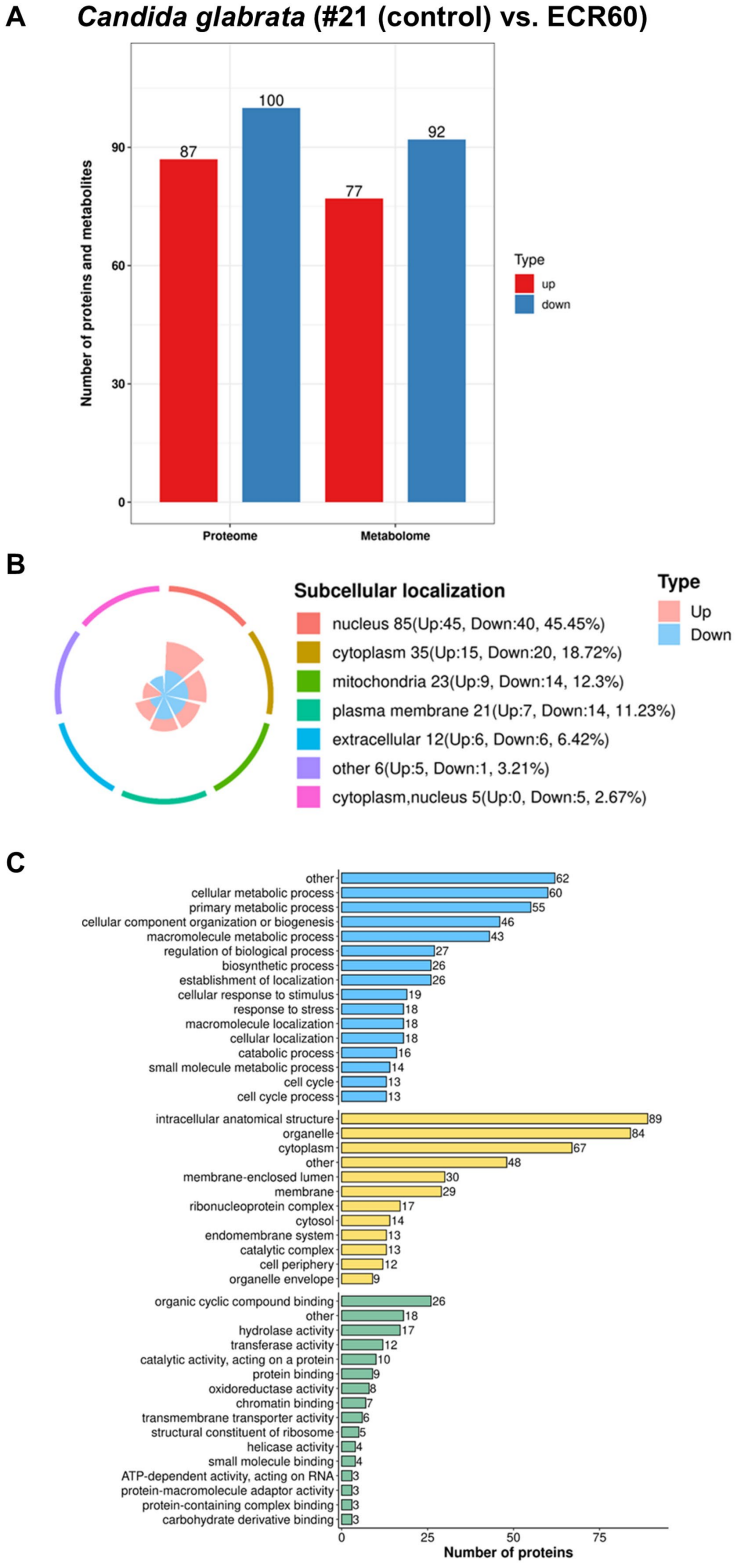
Analysis of protein and metabolite profiles in echinocandin tolerant isolate #21 vs. echinocandin resistant isolate ECR60 strains of *Candida glabrata*. **(A)** Bar charts illustrate the expression levels of proteins and metabolites: proteins that are up-regulated (in red) exhibit a ratio > 1.5 with a significance of *p* < 0.05, while down-regulated proteins (in blue) have a ratio < 0.67 and *p* < 0.05. Additionally, metabolites are represented with a VIP > 1 and *p* < 0.05. **(B)** Visualization of the subcellular localization for these differentially expressed proteins. **(C)** Gene Ontology functional enrichment analysis categorizing the down-regulated proteins.

To explore the mitochondrial changes in echinocandin resistant *C. glabrata*, we further selected the echinocandin tolerant *C. glabrata* #21 and echinocandin resistant *C. glabrata* ECR60. ECR60 was induced by the effect of echinocandin in animals infected with #21 (Supplementary Data 3) (Cai et al., 2026). Importantly, both strains demonstrate a non-mutated echinocandin *FKS* gene (Supplementary Data 1). Through quantitative proteomics, we identified 87 upregulated proteins and 100 downregulated proteins in the ECR60 compared to the #21. Our untargeted metabolomics analysis revealed 77 upregulated and 92 downregulated metabolites (Figure 3A; Supplementary Data 4). Additionally, we assessed the subcellular localization of differentially expressed proteins, focusing on those associated with mitochondria, plasma membranes, and nuclei, as well as those present in both cytoplasm and nucleus (Figure 3B). Significant alterations were noted in mitochondrial proteins, with 9 proteins exhibiting increased expression and 14 showing decreased expression (Supplementary Table S2). Compared with the echinocandin tolerant isolate #21, the echinocandin resistant isolate ECR60 without *FKS* mutation showed a lower the ATP level (Figure 2A). This reduction likely correlates with the decreased representation of ATP-dependent activity in the GO functional annotation of ECR60 (Figure 3C). Notably, membrane potential and intracellular ROS levels showed no significant variation between the two strains (Figures 2B,D). These findings once again highlight the distinct molecular and metabolic adaptations involved in echinocandin resistance in *C. glabrata*, particularly highlighting mitochondrial function. The altered expression of mitochondrial proteins, alongside changes in ATP levels and ROS, suggests critical pathways may be involved in resistance mechanisms.

### 3.3 Comparative proteomics and metabolomics of echinocandin susceptible *Candida glabrata* vs. echinocandin resistant *Candida glabrata* with CRS-MIS phenotype and no *FKS* mutation (CBS138 vs. ECR60)

**Figure 4:**
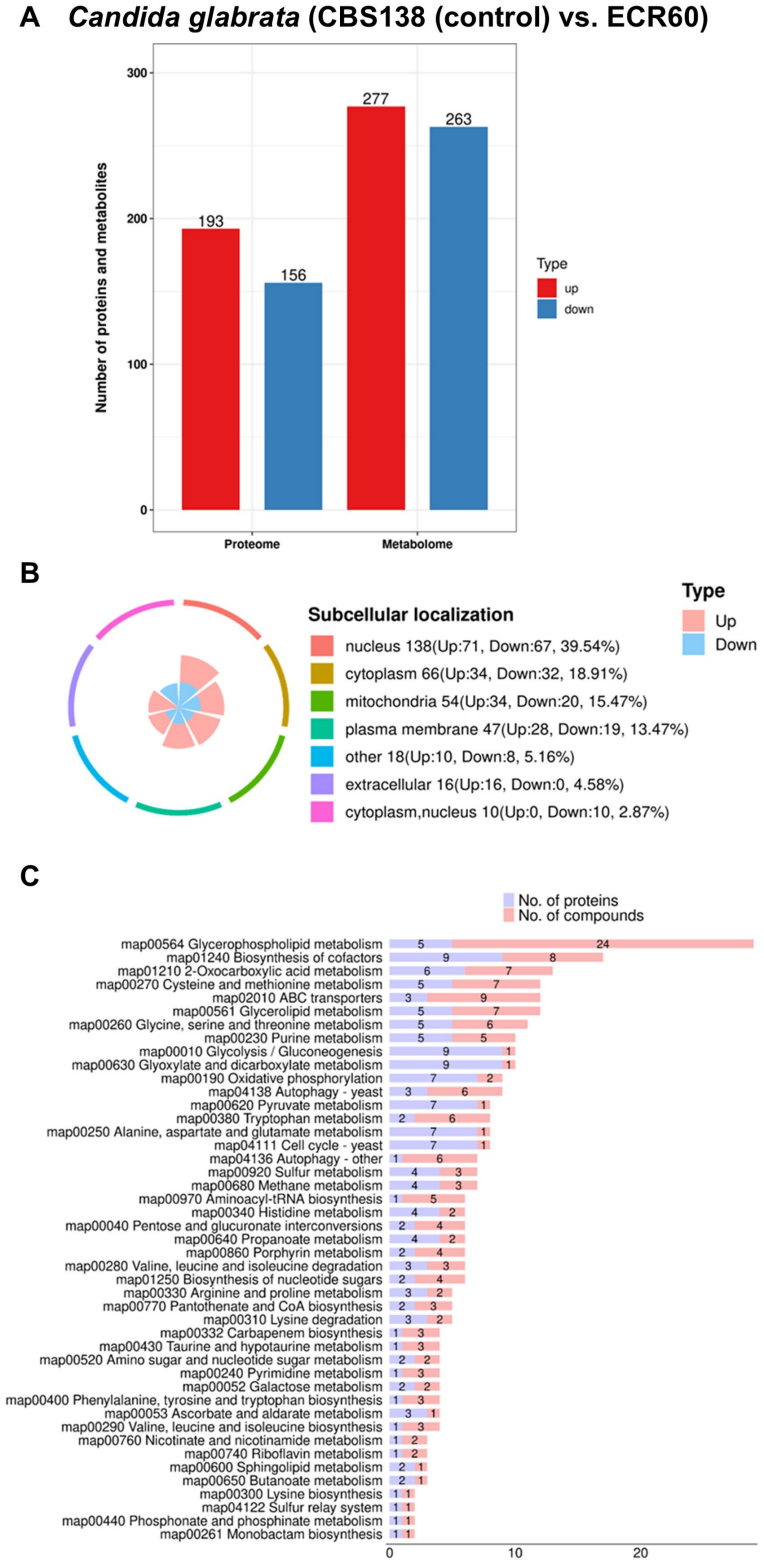
Comparison of protein and metabolite profiles of the echinocandin susceptible strain CBS138 vs. the echinocandin resistant isolate ECR60 in *Candida glabrata*. **(A)** The bar charts illustrate up-regulated proteins (red) with a ratio > 1.5 and *p* < 0.05, alongside down-regulated proteins (blue) with a ratio < 0.67 and *p* < 0.05, and metabolites with VIP > 1 and *p* < 0.05. **(B)** Subcellular localization findings for the differentially expressed proteins are provided. **(C)** Pathway-based classification of the metabolites and proteins along with correlation analyses is exhibited.

In comparing strains CBS138 and ECR60, proteomic analysis revealed that 193 proteins were upregulated while 156 were downregulated in the ECR60 (Figure 4A). Untargeted metabolomics indicated that 277 metabolites were upregulated and 263 downregulated (Figure 4B; Supplementary Data 4). The subcellular localization analysis showed significant changes in mitochondrial proteins, identifying 54 altered proteins (34 upregulated and 20 downregulated) (Supplementary Table S3). The correlation of differential proteins and metabolites again showed that the changes were strongly related to oxidative phosphorylation and purine metabolism (Figure 4C). Investigation results demonstrated increased ATP levels in echinocandin susceptible strains relative to echinocandin resistant ones (CBS138 vs. ECR60), coinciding with a reduction in membrane potential and lower mitochondrial ROS levels, while intracellular ROS presented no significant differences (Figure 2). Collectively, this comparative study emphasizes the mitochondrial characteristics in echinocandin resistant *C. glabrata*.

### 3.4 Comparative proteomics and metabolomics of echinocandin susceptible *Candida glabrata* vs. echinocandin resistant *Candida glabrata* with *FKS* mutation (CBS138 vs. ECR157)

**Figure 5:**
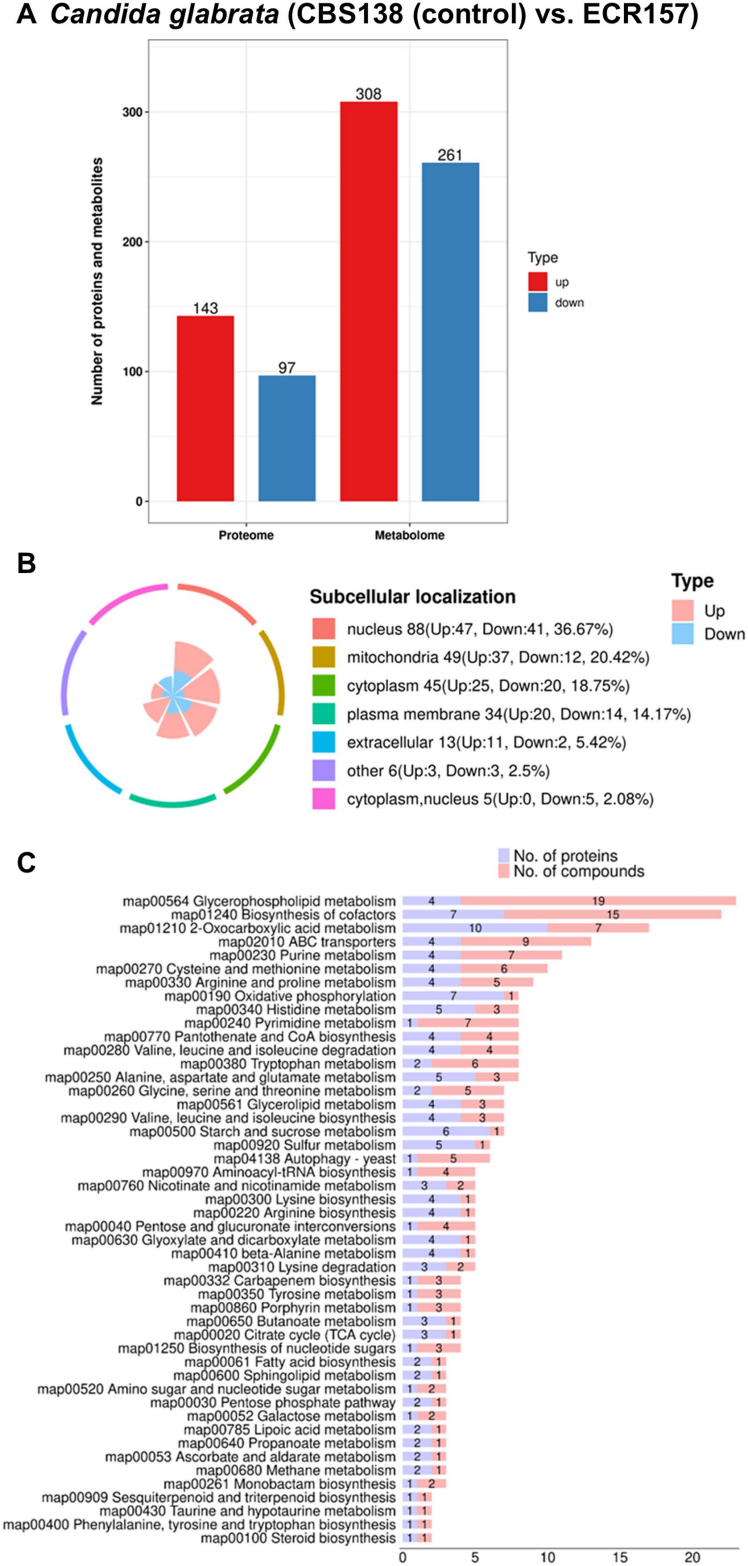
Investigation into the protein and metabolite profiles of echinocandin susceptible strain CBS138 and the echinocandin resistant isolate ECR157 of *Candida glabrata*. **(A)** Differentially expressed proteins and metabolites are represented in bar charts: proteins that are up-regulated (red) with a ratio > 1.5 and *p* < 0.05; down-regulated proteins (blue) with a ratio < 0.67 and *p* < 0.05; and metabolites with VIP > 1 and *p* < 0.05. **(B)** Insights into the subcellular localization of differentially expressed proteins are provided. **(C)** Pathway classification for metabolites and proteins alongside correlation analyses is included.

**Figure 6:**
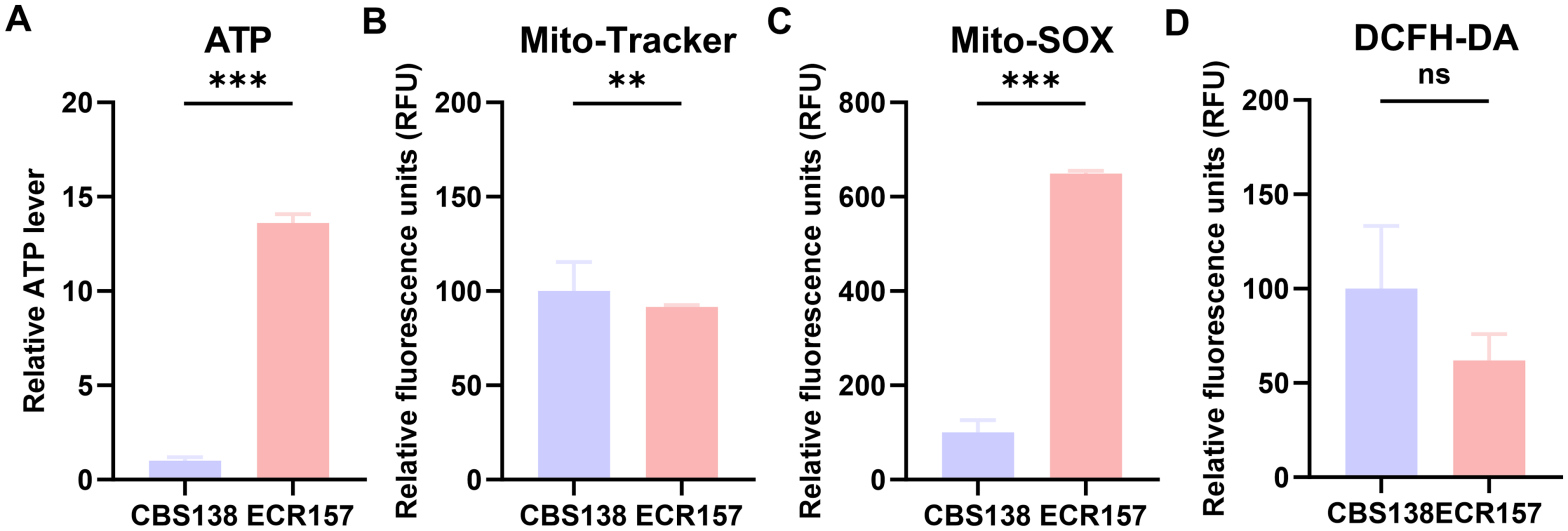
The examination of intracellular ATP levels **(A)**, mitochondrial membrane potential **(B)**, mitochondrial superoxide levels **(C)**, and intracellular ROS levels **(D)** across two *Candida glabrata* strains: echinocandin susceptible strain CBS138 and echinocandin resistant isolate ECR157. Statistical significance was assessed using a t-test, marked by ** (*p* < 0.01), *** (*p* < 0.001), and ns for not significant.

In order to further rule out the effect of CRS-MIS phenotype and *FKS* mutation on the results, we compared echinocandin susceptible *C. glabrata* CBS138 with echinocandin resistant *C. glabrata* ECR157 which was also induced from #21 *in vivo* and has F659del of *FKS* mutation (Supplementary Data 1) (Cai et al., 2026). ECR157 is resistant to both caspofungin and micafungin (Supplementary Data 3). Quantitative proteomic analysis identified 143 upregulated proteins and 97 downregulated proteins (Figure 5A), while untargeted metabolomics revealed 308 upregulated and 261 downregulated metabolites (Figure 5B; Supplementary Data 4). Analysis of mitochondrial proteins revealed significant changes in 49 proteins: 37 were upregulated, and 12 were downregulated (Supplementary Table S4). Correlation analyses pointed to connections with oxidative phosphorylation, purine metabolism, and the tricarboxylic acid (TCA) cycle (Figure 5C). ECR157 displayed elevated ATP levels and mitochondrial ROS, alongside decreased membrane potential. Among them, ECR157 has the highest ATP level among the four strains of *C. glabrata* (Figures 2, 6). These findings further emphasize that echinocandin resistant *C. glabrata* have mitochondrial changes even in the presence of classical *FKS* mutations.

### 3.5 Analysis of genomic variants

**Figure 7:**
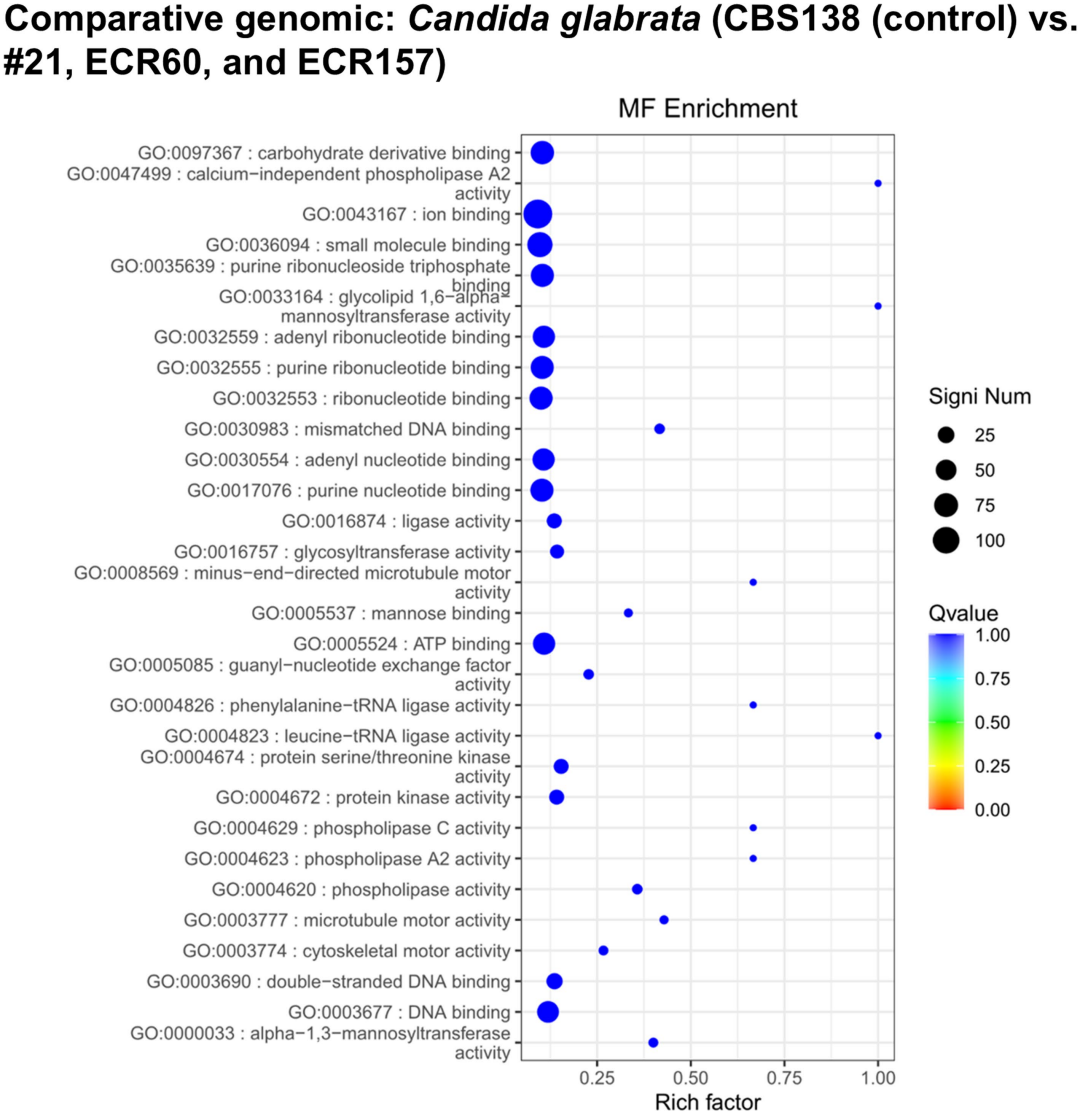
GO molecular function (MF) analysis reveals the enriched functional categories in *Candida glabrata* strains #21, ECR60, and ECR157 relative to the CBS138 control, showing the top 30 enriched GO terms along with their significance. Enriched MF GO terms are shown on the y-axis. Circle size represents the number of significantly enriched genes (Signi Num) per term. Circle color indicates the Q-value. The x-axis represents the rich factor.

To validate this association at a more fundamental genetic level, MF of GO term enrichment analysis was performed using the echinocandin susceptible *C. glabrata* CBS138 as a control, comparing it with the tolerant isolate #21, the resistant isolate ECR60 (without *FKS* mutation), and the resistant isolate ECR157 (with *FKS* mutation) of *C. glabrata* (Supplementary Data 1). The results showed that all three isolates with distinct characteristics exhibited consistent enrichment in multiple functional categories related to ATP binding and protein kinase activity, suggesting that widespread reprogramming of energy metabolism and signal transduction pathways may be involved in the adaptive evolution of *C. glabrata* to echinocandins. Furthermore, phospholipase C and phospholipase A2 activities, which are regulated by mitochondrial functional status, were also significantly enriched, indicating that mitochondria in echinocandin resistant/tolerant isolates may be in a state of sustained stress or functional remodeling. Besides, although #21, ECR60, and ECR157 differed in their tolerant/resistant phenotypes and genotypes, all three showed enrichments in glycosyltransferase activity-related terms, whereas the echinocandin susceptible *C. glabrata* CBS138 did not exhibit similar changes in these pathways (Figure 7).

### 3.6 Mitochondrial characteristics in echinocandin susceptible *Candida krusei* vs. echinocandin resistant *Candida krusei* (ATCC6258 vs. CK225)

**Figure 8:**
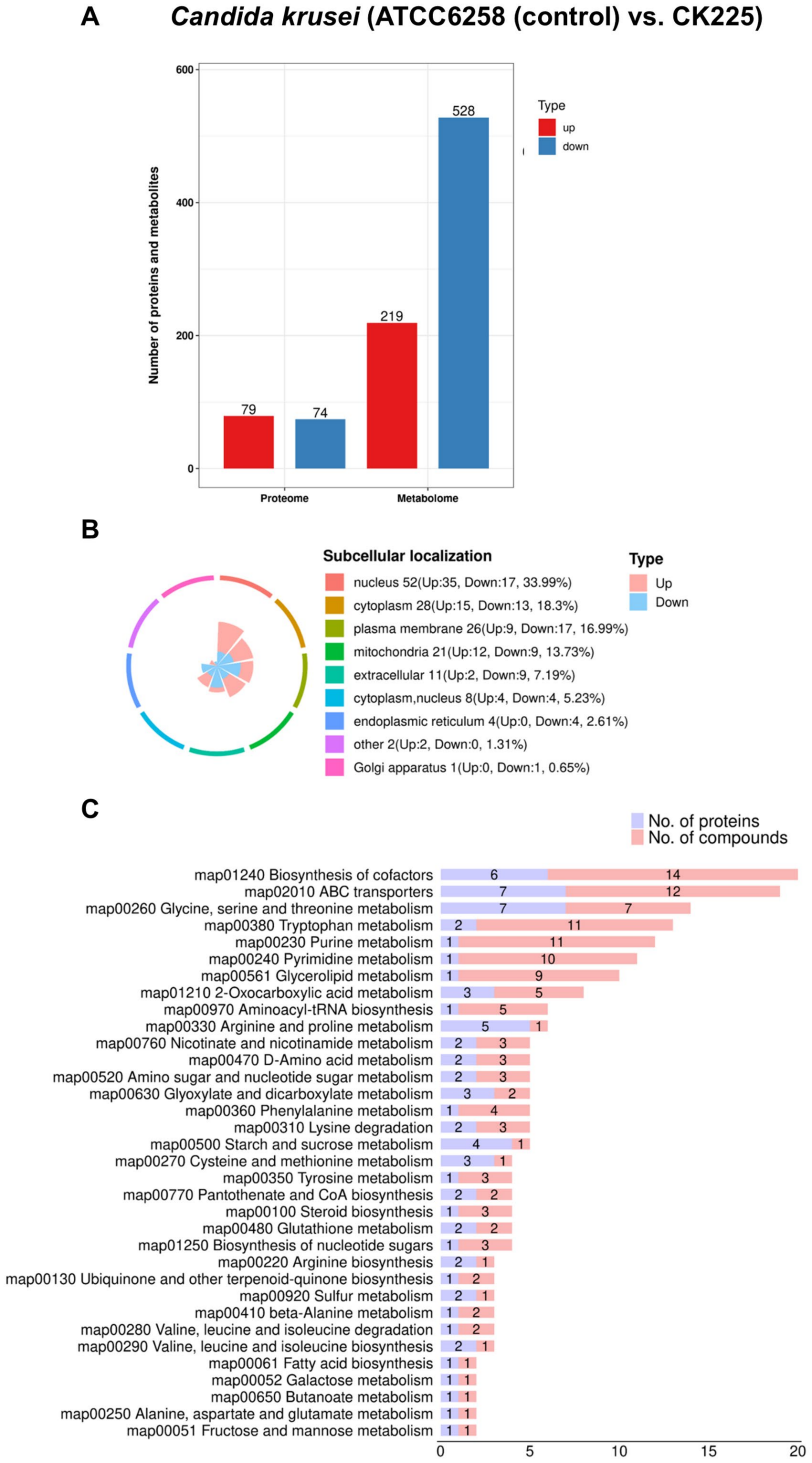
Analysis of protein and metabolite profiles in echinocandin susceptible strain ATCC6258 and echinocandin resistant isolate CK225 strains of *Candida krusei*. **(A)** Differentially expressed proteins and metabolites are presented in bar charts: up-regulated proteins (red; ratio > 1.5, *p* < 0.05), down-regulated proteins (blue; ratio < 0.67, *p* < 0.05), and metabolites with VIP > 1 and *p* < 0.05. **(B)** Subcellular localization of differentially expressed proteins is depicted. **(C)** Classification of pathways for metabolites and proteins, along with correlation analyses, is provided.

**Figure 9:**
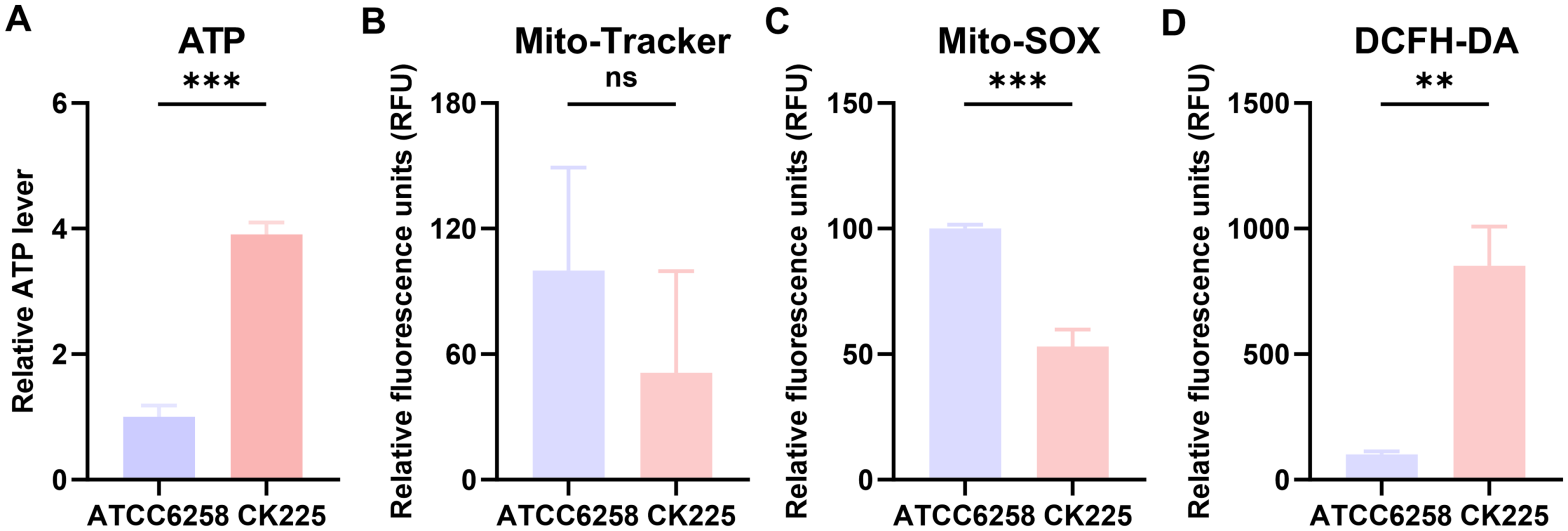
Assessment of intracellular ATP levels **(A)**, mitochondrial membrane potential **(B)**, mitochondrial superoxide levels **(C)**, and intracellular ROS levels **(D)** in two *Candida krusei* strains: echinocandin susceptible strain ATCC6258 and echinocandin resistant isolate CK225. Statistical significance was determined using a t-test, with results marked as ** (*p* < 0.01), *** (*p* < 0.001), and ns indicating not significant.

**Figure 10:**
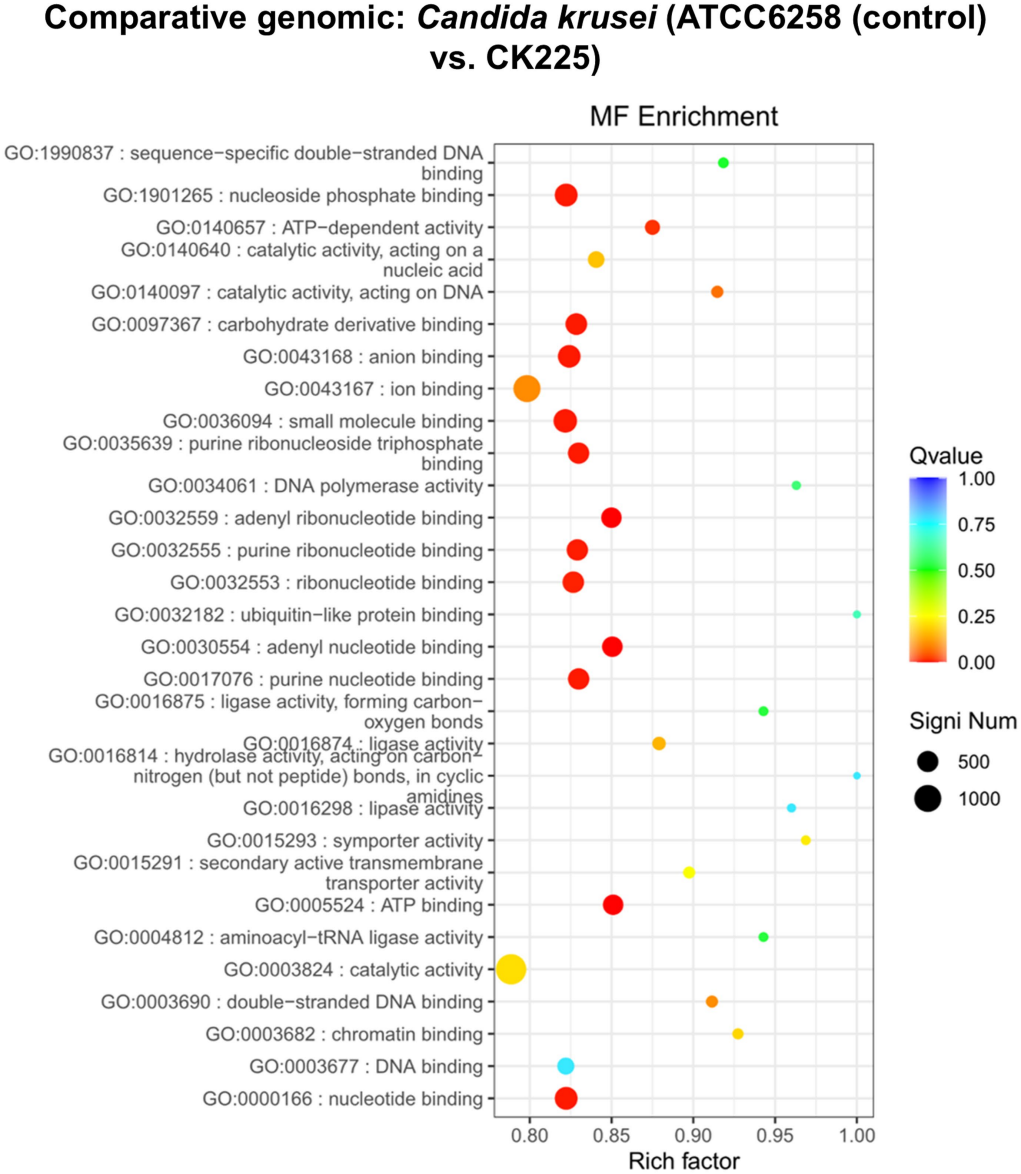
Enrichment analysis of GO molecular functions for the *Candida krusei* strains ATCC6258 and CK225 genomes, highlighting the top 30 enriched GO terms along with their corresponding significance levels. The X-axis represents enriched factors. The Y-axis represents rich MF GO terms. The circle size indicates the number of significantly enriched genes (Signi Num) for each term. The circle color indicates the Q value.

To expand our understanding of the mitochondrial characteristics in different echinocandin sensitivity of *Candida*, we analyzed echinocandin susceptible standard strain ATCC6258 against the echinocandin resistant isolate CK225 of *C. krusei*. Quantitative proteomic analysis revealed 79 upregulated proteins and 74 downregulated proteins in the CK225, reinforced by untargeted metabolomics that identified 219 upregulated and 528 downregulated metabolites (Figure 8A; Supplementary Data 4). Differential analysis of mitochondrial proteins indicated changes in 21 proteins: 12 were upregulated, and 9 were downregulated (Figure 8B; Supplementary Table S5). Correlation analyses of these differential proteins and metabolites indicated a close association with purine metabolism, demonstrating the adaptive strategies employed by *C. krusei* in response to echinocandin resistance (Figure 8C). The results of mitochondrial related phenotypes also indicated a significant increase in ATP levels and intracellular ROS levels (Figures 9A,D). Interestingly, mitochondrial ROS levels were significantly lower (Figure 9C), and there were no notable changes in membrane potential (Figure 9B). Furthermore, Figure 10 outlines the GO analysis for the genomes of *C. krusei* ATCC6258 and CK225, detailing the top 30 enriched GO terms. ATP binding and mitochondrial associated activity pathways were also enriched. Collectively, the role of mitochondria in altering echinocandin susceptibility in *Candida* species extends from *C. glabrata* to *C. krusei*, confirming a certain degree of universality.

## 4 Discussion

This study provided comprehensive evidence revealing the mitochondrial characteristics in different echinocandin sensitivity *Candida* from four levels: mitochondrial phenotype, proteomics, metabolomics, and genomics. The reliability of the results was enhanced, and the applicability of the results was expanded through the use of two strains, *C. glabrata* and *C. krusei*. The results showed that, using the echinocandin sensitive standard strain as a reference control, regardless of *C. glabrata* or *C. krusei*, whether tolerant or resistant to echinocandin, *Candida* showed high ATP levels. This trend is consistent with the observation of azole resistant strains, indicating that the effect of mitochondria on antifungal drug action can be extended to echinocandin (Zhou et al., 2024). This result suggests that ATP generated by fungal high metabolism is expected to provide critical energy support for these echinocandin tolerant and resistant adaptation changes. Interestingly, the echinocandin resistant *C. glabrata* ECR157 with *FKS* mutations exhibited the highest ATP levels. The ATP level of the echinocandin resistant *C. glabrata* ECR60 without *FKS* mutation is lower than that of #21, although both still maintain high ATP levels relative to CBS138. This suggests that ECR60 may adopt other compensatory mechanisms to ensure sufficient energy production during drug exposure.

Antifungal drugs, including echinocandins, azoles, and polyenes, have been shown to induce ROS through mitochondria and promote the adaptive evolution of fungal resistant to antifungal drugs (Gonzalez-Jimenez et al., 2023). Compared with the echinocandin sensitive *C. glabrata* CBS138, the mitochondrial ROS of the tolerant *C. glabrata* #21 and the resistant *C. glabrata* ECR157 with *FKS* mutations were significantly increased. This result is consistent with previous research that echinocandin resistant *Aspergillus fumigatus* with *FKS* mutation shows ROS increase (Satish and Perlin, 2019). This study extends mitochondrial characteristics of echinocandin sensitivity from filamentous fungi to *Candida*.

However, the mitochondrial ROS levels in ECR60 were significantly lower than those in #21 and CBS138. This suggests that although some strains, especially those with *FKS* mutations, may rely on elevated mitochondrial ROS levels to achieve resistant to antifungal drugs, strains without *FKS* mutations such as ECR60 may evolve another non classical adaptive compensatory change mechanism that relies on ATP levels, reducing oxidative stress by lowering ROS levels and regulating ATP levels to ensure the production of appropriate energy to maintain balance and survival in antifungal drugs exposure environments. For example, previous studies have shown that echinocandin tolerant strains have significantly lower basal ATP levels than their parent strains due to mitochondrial functional defects. However, after macrophage internalization, the ATP levels of tolerant strains exhibit dynamic changes, with lower levels in the early stages and higher levels in the later stages. This may be due to compensatory upregulation of host cell ATP supply or glycolysis pathways. And the consistent trend of mitochondrial membrane potential decline exhibited by the echinocandin tolerant and resistant strains involved in our study is also consistent with the findings of the above-mentioned study (Arastehfar et al., 2023). Meanwhile, this discovery has also been validated in azole resistant *Candida*, where when mitochondrial functional efficiency slightly decreases (membrane potential decreases), *Candida* can produce a large amount of ATP by enhancing glycolysis compensation, maintaining or even increasing overall energy levels (Zhou et al., 2024).

In terms of intracellular ROS levels, there was no significant difference among strains sensitive, tolerant, and resistant to echinocandin, indicating that intracellular ROS levels may fluctuate between strains with different drug sensitivities, but overall remain within a stable range. In summary, the results of the above four indicators suggest that the adaptive evolution of *Candida*’s tolerant/resistant to echinocandin may be related to mitochondrial changes, but further research is needed to prove it. Besides, the specific mitochondrial characteristics of *Candida* may serve as early biomarkers for predicting and rapidly identifying echinocandin tolerance/resistance, thereby guiding the clinical use of antifungal drugs.

This study combines multiple omics (proteomics, metabolomics, genomics) to further systematically analyze the changes in energy metabolites, mitochondrial protein expression, and genes related to *Candida* mitochondrial function and sensitivity to echinocandin from sequencing to phenotype, deepening the understanding of mitochondrial characteristics in echinocandin tolerant/resistant *Candida*. Mitochondrial function is not only crucial for energy metabolism, but also plays a key role in regulating oxidative stress response, which is an important component of drug resistant mechanisms. The balance between energy metabolism and oxidative stress regulation may be a key strategy for drug resistant strains to survive under antifungal drug pressure. Our result is also similar to previous studies, where transcriptome analysis found that multiple mitochondrial function related genes were downregulated in echinocandin tolerant strains, but at the same time, ATP binding related functional proteins were significantly enriched (Garcia-Rubio et al., 2021). Therefore, based on the results of our study, echinocandin tolerant/resistant *Candida* may have dynamic energy adaptation patterns, which may be balanced between energy demand and oxidative damage avoidance through metabolic reprogramming, achieving survival under echinocandin pressure. However, this hypothesis is currently only supported by omics data and requires further experimental evidence.

This study focused on common pathogens in candidemia, such as *C. glabrata* and *C. krusei*, and comprehensively revealed the mitochondrial characteristics of echinocandin sensitivity among different *Candida* species. Although the strain design of this study was proved by previous studies (Zhou et al., 2024) and utilized multiple *Candida* species types, with two isolates from the same *Candida* species, the results still require validation in a larger panel of strains. On one hand, it is necessary to increase the number of strains to enhance the credibility of the findings; on the other hand, it is essential to broaden the range of *Candida* species, including other *Candida* species or filamentous fungi, to verify the generalizability of the mitochondrial changes observed. In the future, if more filamentous fungi are included for more extensive studies, it may help clarify the role of mitochondria in the adaptive evolution of antifungal drug tolerance/resistance across fungi. Future research may also consider including strains with different resistant profiles, such as single polyene or fluorocytosine resistance, or multidrug-resistant fungal, to confirm the universality of mitochondrial characteristics in different fungis. The application of new technologies, such as super-resolution microscopy, also contributes to a deeper understanding of the association between *Candida* drug sensitivity and mitochondria (Li et al., 2024).

In summary, the adaptive evolution of *Candida* tolerance or resistance has increased the complexity of clinical antifungal treatment (Tang et al., 2025). The emergence of *Candida* with non-classical drug resistant mechanisms has further increased the difficulty of antifungal drug resistant management (Dong et al., 2026). Our study revealed significant differences in gene composition, protein expression, metabolite profile, and mitochondrial phenotype among *Candida* species with different levels of sensitivity to echinocandin, suggesting that mitochondrial function plays an important role in the survival of *Candida* under echinocandin pressure. These findings provide new insights into how *Candida* resists killing through mitochondrial related adaptive changes under antifungal drug pressure, and are expected to further explore new ways to enhance antifungal drug activity or prevent the evolution of *Candida* tolerance and resistance adaptation.

## Data Availability

The genomics data are accessible in the GSA database under accession number CRA044057 (https://ngdc.cncb.ac.cn/gsa). The proteomics and metabolomics data can be found in the OMIX database, with accession numbers OMIX017469 (https://ngdc.cncb.ac.cn/omix/release/OMIX017469) and OMIX017386 (https://ngdc.cncb.ac.cn/omix/release/OMIX017386), respectively.
