# Supplementary material for: Mitochondrial characteristics of echinocandin tolerance and resistance in Candida glabrata and Candida krusei
Source: Front Microbiol. 2026 Jul 27;17:1906168. doi: 10.3389/fmicb.2026.1906168 (PMC13454047; doi:10.3389/fmicb.2026.1906168)
Supplement: Supplementary file 1 [file Data_Sheet_5.PDF]

Supplementary Material

1 Supplementary Methods

1.1 Sample preparation

Lysis buffer contained 10 mM dithiothreitol, 1% Triton X-100, protease inhibitor cocktail, 50 mM NAM, 50 µM PR-619, 2 mM EDTA, 3 µM TSA, and 1% phosphatase inhibitor. The suspension was sonicated for 3 min on ice. Proteins were extracted with Tris-saturated phenol (pH 8.0, Sigma-Aldrich), vortexed for 5 min, and centrifuged at 4°C for 10 min. The phenolic phase was precipitated with ≥4 volumes of ammonium sulfate-saturated methanol at -20°C for ≥6 h. The pellet was washed with ice-cold methanol and acetone (×3), then solubilized in 8 M urea. Protein concentration was determined using a BCA assay kit (Beyotime). For further purification, TCA was added to a final concentration of 20% and incubated at 4°C for 2 h. The pellet was washed, briefly dried, and re-dissolved in 200 mM TEAB (Sigma-Aldrich) via ultrasonic dispersion. Proteins were reduced with 5 mM DTT at 56°C for 30 min, alkylated with 11 mM iodoacetamide in the dark for 15 min, and digested overnight with trypsin (1 µg/µL in 50 mM acetic acid) at an enzyme-to-protein ratio of 1:50 (w/w). Peptides were desalted using a Strata X SPE column (Phenomenex).

1.2 LC-MS/MS analysis

Solvent A: 0.1% formic acid, 2% acetonitrile; solvent B: 0.1% formic acid in acetonitrile. The gradient was: 6–24% B over 14 min, 24–35% B over 2 min, 35–90% B over 2 min, and held at 90% B for 2 min, at a flow rate of 500 nL/min. Separation was performed on a custom-made 25 cm reversed-phase column (100 µm i.d.) using an Easy-nLC1000 UHPLC system (Bruker Daltonics). The mass spectrometer (timsTOF Pro, Bruker) was operated in dia-PASEF mode with an ESI voltage of 1.75 kV, acquiring 20 PASEF-MS/MS scans per cycle (MS range 300–1500 m/z; MS/MS range 400–850 m/z) with a 7 m/z isolation window.

1.3 Data processing

DIA-NN (v1.8) was used for data processing. For *Candida glabrata*, the XC08588.fasta database (5,145 entries) was used; for *Candida krusei* (*Pichia kudriavzevii*), the Pichia_kudriavzevii_4909_PR_20241023.fasta database (5,146 entries) was used, both supplemented with reverse decoys. Trypsin/P was specified with one missed cleavage allowed. Fixed modifications included N-terminal methionine removal and carbamidomethylation of cysteine. The false discovery rate (FDR) was set to 1% (strict). Gene Ontology (GO) annotation was performed using eggnog-mapper v2.1.12 against the EggNOG database. KEGG pathway annotation was obtained via BLASTP (e-value $\leq 10^{-4}$) against the KEGG database, selecting the top hit. Subcellular localization was predicted using WolF PSORT. Functional enrichment was assessed using Fisher's exact test, with the full identified protein set as background; terms with a fold enrichment > 1.5 and $p < 0.05$ were considered significant.

2 Supplementary Tables

Table S1 Mitochondrial proteins were evaluated for differential expression between the control strain *Candida glabrata* CBS138 and strain #21. Significant differences were found in proteins with a ratio (#21/CBS138) demonstrating a fold change greater than 1.5 or less than 0.67, along with a *p*-value below 0.05.

Protein description	Gene name	#21/CBS138 Ratio
Pore membrane protein of 33 kDa	AO440_001643	2.44
L37	AO440_003964	1.90
Peroxisomal long-chain fatty acid import protein 1	AO440_000622	1.85
Glutaredoxin-2, mitochondrial	AO440_003501	1.56
Isocitrate dehydrogenase [NAD] subunit 2	AO440_002646	1.65
Ferrochelatase	AO440_004635	1.86
ATP synthase subunit 5	AO440_001959	1.51
Mitochondrial ATPase complex subunit ATP10	AO440_000507	1.68
Protein SYM1	AO440_000308	2.30
Uncharacterized protein	AO440_004164	1.64
Large ribosomal subunit protein uL30m	AO440_002453	1.54
37S ribosomal protein S26	AO440_000467	1.54
ABC1 family protein MCP2	AO440_000307	1.51
Uncharacterized protein	AO440_004893	3.81
Altered inheritance of mitochondria protein 19	AO440_004139	1.54
Succinate/fumarate mitochondrial transporter	AO440_004240	1.58

Protein description	Gene name	#21/CBS138 Ratio
NADH:ubiquinone reductase (non-electrogenic)	AO440_002374	1.80
Small ribosomal subunit protein uS11m	AO440_004023	1.51
Mitochondrial metal transporter 2	AO440_001082	1.95
D-lactate dehydrogenase (cytochrome)	AO440_002558	3.29
L-lactate dehydrogenase (cytochrome)	AO440_003702	3.07
Peroxisomal hydratase-dehydrogenase-epimerase	AO440_004484	1.88
MICOS complex subunit MIC10	AO440_000189	1.89
Mitochondrial escape protein 2	AO440_004444	1.65
ATP synthase subunit beta	AO440_001920	1.56
Enoyl-[acyl-carrier-protein] reductase	AO440_000346	1.54
Ribosome-recycling factor	AO440_001476	1.52
Heat shock protein 78	AO440_004232	1.59
ATP-dependent (S)-NAD(P)H-hydrate dehydratase	AO440_001098	2.04
dihydrolipoyllysine-residue succinyltransferase	AO440_000929	1.63
Tyrosine--tRNA ligase	AO440_002140	1.73
Putative aryl-alcohol dehydrogenase	AO440_001041	0.42
Putative ABC transporter ATP-binding protein	AO440_001693	0.62
C-5 sterol desaturase	erg3	0.49
Adenylyl-sulfate kinase	AO440_004477	0.46

Protein description	Gene name	#21/CBS138 Ratio
homoisocitrate dehydrogenase	AO440_001591	0.50
Aconitate hydratase	AO440_001201	0.46
Uncharacterized protein	AO440_001407	0.44
Proline dehydrogenase	AO440_004051	0.65
Protein KES1	AO440_004876	0.24
Homoaconitase	AO440_003713	0.61
NEDD8-conjugating enzyme UBC12	AO440_000876	0.59
Branched-chain-amino-acid aminotransferase	AO440_001488	0.61
Aminomethyltransferase	AO440_000797	0.66
dihydroxy-acid dehydratase	AO440_000332	0.53

Table S2 Mitochondrial proteins were were analyzed for differential expression between the control strain *Candida glabrata* #21 and strain ECR60. Significant differences were found in proteins with a ratio (ECR60/#21) showing a fold change greater than 1.5 or less than 0.67, and a *p*-value under 0.05.

Protein description	Gene name	ECE60/#21 Ratio
Large ribosomal subunit protein mL53	AO440_004337	1.77
60S ribosomal protein L43	AO440_002270	1.66
C-5 sterol desaturase	erg3	1.59
Protein KES1	AO440_004876	2.57
N-terminal acetyltransferase	AO440_005589	1.55
Monothiol glutaredoxin-5	AO440_004167	1.77
Sulfhydryl oxidase	AO440_003482	1.61
L37	AO440_003964	20.95
tRNA pseudouridine(27/28) synthase	AO440_004104	1.51
COX3 mRNA-specific translational activator PET494	AO440_000544	0.63
Thioredoxin-3	AO440_000899	0.58
37S ribosomal protein S26	AO440_000467	0.61
54S ribosomal protein L16	AO440_001283	0.66
Protein SYM1	AO440_000308	0.58
Putative abhydrolase domain-containing protein	AO440_001474	0.41
Pre-mRNA-splicing factor SAD1	AO440_005210	0.53
Citrate synthase	AO440_005206	0.61
Large ribosomal subunit protein Ul23	AO440_004022	0.54

Protein description	Gene name	ECE60/#21 Ratio
Acyl carrier protein	AO440_000764	0.48
Nascent polypeptide-associated complex subunit beta	AO440_004835	0.64
Cytochrome c oxidase subunit 4	AO440_005683	0.60
GTP-binding protein	AO440_000268	0.60
5-aminolevulinate synthase	AO440_000275	0.63

Table S3 Mitochondrial proteins were analyzed for differential expression between the *Candida glabrata* CBS138 control strain and strain ECR60. Significant differences were observed in proteins with a ratio (ECR60/CBS138) showing a fold change greater than 1.5 or less than 0.67, and a *p*-value of less than 0.05 indicating statistical significance.

Protein description	Gene name	ECR60/CBS138 Ratio
Ferrochelatase	AO440_004635	1.73
37S ribosomal protein S12	AO440_001812	1.57
37S ribosomal protein RSM28	AO440_001675	1.50
ATP synthase subunit d	AO440_005228	1.69
SURF1-like protein	AO440_004638	1.56
L37	AO440_003964	39.74
DBF2 kinase activator protein MOB1	AO440_001601	1.64
[acyl-carrier-protein] S-malonyltransferase	AO440_004885	1.51
Large ribosomal subunit protein Ml53	AO440_004337	1.83
Uncharacterized protein	AO440_004893	4.76
Mitochondrial ATPase complex subunit ATP10	AO440_000507	1.58
37S ribosomal protein MRP21	AO440_002085	1.52
Uncharacterized protein	AO440_004164	1.68
60S ribosomal protein L43	AO440_002270	1.72
Large ribosomal subunit protein uL3m	AO440_000255	1.98
Altered inheritance of mitochondria protein 19	AO440_004139	1.84
37S ribosomal protein S16	AO440_005191	1.51

Protein description	Gene name	ECR60/CBS138 Ratio
Pore membrane protein of 33 kDa	AO440_001643	1.89
RNA exonuclease NGL1	AO440_001145	1.52
ATP synthase subunit 5	AO440_001959	1.84
Large ribosomal subunit protein uL30m	AO440_002453	1.99
Succinate dehydrogenase [ubiquinone] iron-sulfur subunit	AO440_000530	1.61
Large ribosomal subunit protein uL13m	AO440_004614	1.50
NADH:ubiquinone reductase (non-electrogenic)	AO440_002374	1.60
ATP synthase subunit beta	AO440_001920	1.64
L-lactate dehydrogenase (cytochrome)	AO440_003702	2.80
MICOS complex subunit MIC10	AO440_000189	1.68
Glutamate pyruvate transaminase	AO440_004823	1.63
N-acetyltransferase ECO1	AO440_002723	1.78
ATP-dependent (S)-NAD(P)H-hydrate dehydratase	AO440_001098	1.61
Tyrosine--tRNA ligase	AO440_002140	1.63
Mitochondrial metal transporter 2	AO440_001082	1.63
D-lactate dehydrogenase (cytochrome)	AO440_002558	2.28
Heat shock protein 78, mitochondrial	AO440_004232	1.57
tRNA pseudouridine(31) synthase	AO440_002713	0.38
Large ribosomal subunit protein uL23	AO440_004022	0.60

Protein description	Gene name	ECR60/CBS138 Ratio
5-aminolevulinate synthase	AO440_000275	0.56
Protein translocation protein SEC63	AO440_001790	0.62
Intron-encoded DNA endonuclease aI5 alpha	AO440_004680	0.66
Uncharacterized protein	AO440_001407	0.64
Pre-mRNA-splicing factor SAD1	AO440_005210	0.66
Mitochondrial thiamine pyrophosphate carrier 1	AO440_001614	0.51
Putative bolA-like protein	AO440_004379	0.66
Aconitate hydratase	AO440_001201	0.66
Uncharacterized hydrolase	AO440_002650	0.41
Putative aryl-alcohol dehydrogenase	AO440_001041	0.45
Acyl carrier protein	AO440_000764	0.46
Citrate synthase	AO440_000317	0.66
Nascent polypeptide-associated complex subunit beta	AO440_004835	0.50
Citrate synthase	AO440_005206	0.52
Ran-specific GTPase-activating protein 1	AO440_000453	0.65
Aminomethyltransferase	AO440_000797	0.66
GTP-binding protein	AO440_000268	0.56
Adenylyl-sulfate kinase	AO440_004477	0.51

Table S4 Mitochondrial proteins were analyzed for differential expression between the *Candida glabrata* CBS138 control strain and strain ECR157. Significant differences were identified in proteins displaying a fold change (ECR157/CBS138) greater than 1.5 or less than 0.67, with a *p*-value below 0.05 indicating statistical significance.

Protein description	Gene name	ECR157/CBS138 Ratio
Isocitrate dehydrogenase [NAD] subunit 2	AO440_002646	1.82
Peptide-methionine (R)-S-oxide reductase	AO440_000157	1.55
ATP synthase subunit d, mitochondrial	AO440_005228	1.77
[acyl-carrier-protein] S-malonyltransferase	AO440_004885	1.54
Protein SCO2	AO440_000348	1.54
Ferrochelatase	AO440_004635	1.87
ATP synthase subunit epsilon	AO440_000043	1.68
ATP synthase subunit 5	AO440_001959	1.66
54S ribosomal protein L22	AO440_003245	1.50
Mitochondrial ATPase complex subunit ATP10	AO440_000507	1.52
Protein SYM1	AO440_000308	2.34
Putative redox protein FMP46	AO440_004110	1.78
Large ribosomal subunit protein uL3m	AO440_000255	1.75
37S ribosomal protein S16	AO440_005191	1.53
Glutaredoxin-2	AO440_003501	1.75
Uncharacterized protein	AO440_004893	4.14
Peroxisomal adenine nucleotide transporter 1	AO440_000813	1.66

Protein description	Gene name	ECR157/CBS138 Ratio
Succinate/fumarate mitochondrial transporter	AO440_004240	1.69
Peroxisomal long-chain fatty acid import protein 1	AO440_000622	1.58
Altered inheritance of mitochondria protein 19	AO440_004139	2.37
Pore membrane protein of 33 kDa	AO440_001643	2.27
SURF1-like protein	AO440_004638	1.57
ATP synthase subunit beta	AO440_001920	1.73
L-lactate dehydrogenase (cytochrome)	AO440_003702	3.08
Mitochondrial escape protein 2	AO440_004444	1.54
Acyl carrier protein	AO440_002966	1.57
Enoyl-[acyl-carrier-protein] reductase	AO440_000346	1.66
ATP-dependent (S)-NAD(P)H-hydrate dehydratase	AO440_001098	1.71
MICOS complex subunit MIC10	AO440_000189	1.67
NADH:ubiquinone reductase (non-electrogenic)	AO440_000267	1.51
Mitochondrial metal transporter 2	AO440_001082	1.77
D-lactate dehydrogenase (cytochrome)	AO440_002558	2.72
Protein SCO1, mitochondrial	AO440_002542	1.52
NADH:ubiquinone reductase (non-electrogenic)	AO440_002374	1.63
Large ribosomal subunit protein uL30m	AO440_002453	2.09
Tyrosine--tRNA ligase	AO440_002140	1.93

Protein description	Gene name	ECR157/CBS138 Ratio
2-oxoglutarate dehydrogenase	AO440_001843	1.67
Protein MNE1	AO440_000705	0.66
homoisocitrate dehydrogenase	AO440_001591	0.58
Adenylyl-sulfate kinase	AO440_004477	0.48
Protein KES1	AO440_004876	0.32
Aconitate hydratase	AO440_001201	0.56
Uncharacterized hydrolase	AO440_002650	0.57
Proline dehydrogenase	AO440_004051	0.58
Putative aryl-alcohol dehydrogenase	AO440_001041	0.36
Homoaconitase, mitochondrial	AO440_003713	0.63
Branched-chain-amino-acid aminotransferase	AO440_001488	0.67
Aminomethyltransferase	AO440_000797	0.67
dihydroxy-acid dehydratase	AO440_000332	0.56

Table S5 The analysis of mitochondrial proteins revealed significant differences in expression between the control strain *Candida krusei* ATCC6258 and strain *C. krusei* CK225. Notable changes were found in proteins with a fold change (CK225/ATCC6258) exceeding 1.5 or falling below 0.67, with a *p*-value under 0.05.

Protein description	Gene name	CK225/ ATCC6258 Ratio
Uncharacterized protein	C5L36_0B00780	1.90
Tryptophan synthase beta chain-like PALP domain-containing protein	C5L36_0B00970	1.53
Uncharacterized protein	C5L36_0C09650	1.56
Protein MMF1, mitochondrial	C5L36_0E05260	1.82
HMA domain-containing protein	C5L36_0A05960	2.36
Uncharacterized protein	C5L36_0C03900	1.69
Uncharacterized protein	C5L36_0C10120	1.51
Mitochondrial peroxiredoxin PRX1	BOH78_0316	1.55
Acetylornithine aminotransferase, mitochondrial	C5L36_0B10040	1.64
Threonine dehydratase	C5L36_0A11960	1.52
Mitochondrial ornithine transporter 1	C5L36_0C11580	1.96
Altered inheritance of mitochondria protein 32	BOH78_0698	1.53
GH18 domain-containing protein	C5L36_0A07150	0.35
PPM-type phosphatase domain-containing protein	C5L36_0D02590	0.65
Prokaryotic-type class I peptide chain release factors domain-containing protein	C5L36_0A09820	0.46
Peptidase M3A/M3B catalytic domain-containing protein	C5L36_0B05320	0.57

Protein description	Gene name	CK225/ ATCC6258 Ratio
Glycine cleavage system P protein	C5L36_0C05860	0.51
Aminomethyltransferase	C5L36_0A08520	0.50
Branched-chain-amino-acid aminotransferase	C5L36_0C09980	0.63
Alpha-1,3/1,6-mannosyltransferase ALG2	C5L36_0B10400	0.64
Dynamin-like GTPase MGM1, mitochondrial	C5L36_0A10580	0.61
